# Downregulation of GSK3β by miR-544a to maintain self-renewal ability of lung caner stem cells

**DOI:** 10.3892/ol.2014.2387

**Published:** 2014-07-28

**Authors:** XIAO-MEI MO, HUA-HUI LI, MING LIU, YAN-TUAN LI

**Affiliations:** 1 Key Laboratory of Marine Drugs, Ministry of Education, School of Medicine and Pharmacy, Ocean University of China, Qingdao, Shandong 266003, P.R. China; 2 Pharmacy Department, Qingdao Women and Children Hospital, Qingdao, Shandong 266011, P.R. China; 3 Medical College of Qingdao University, Qingdao, Shandong 266011, P.R. China; 4 Department of Laboratory Medicine of Qingdao Municipal Hospital, Qingdao, Shandong 266011, P.R. China

**Keywords:** non-small cell lung cancer, cancer stem cells, GSK3β, Wnt signaling pathway, miR-544a

## Abstract

In order to study the influence and mechanism of miR-544a on the self-renewal ability of lung cancer stem cells, TargetScan was used to predict the target gene of miR-544a. A luciferase reporter system and western blotting were used to validate the target genes identified by TargetScan. 95C and 95D low and high metastatic human lung cancer cells were transfected with miR-544a, and quantitative polymerase chain reaction (qPCR) was used to verify the miR-544a expression in these two cell lines. Tumor ball (spheroid) suspension culture was use to study the effects of miR-544a on lung cancer stem cells. TargetScan predicted that miR-544a interacted with GSK3β. A luciferase reporter system (F=201.37, P<0.01) and western blot analysis was used to validate that miR-544a could inhibit the expression of GSK3β, while β-catenin and CD133 were significantly increased in miR-544a-overexpressing 95C and 95D cells (F=9.43, 7.73 and 3.37, respectively; P<0.01). qPCR revealed that miR-544a was overexpressed in transfected 95C and 95D cells (20.51±0.97 and 15.16±1.38, respectively; F=418.05; P<0.01). miR-544a-overexpressing cells formed spheroids in suspension cultures of spheroid single cells. miR-544a was shown to reduce the expression of GSK3β and activate the Wnt signaling pathway to maintain the self-renewal ability of lung caner stem cells.

## Introduction

Tumor metastasis can occur despite radiation and chemotherapy treatment. Non-small cell lung carcinoma (NSCLC) comprises 80% of all types of lung cancer, and a number of cancer patients succumb to cancer metastasis (1). Therefore, further investigation with regard to the mechanism of metastasis in NSCLC is required. A previous study has shown that tumor stem cells (TSC) may be responsible for cancer recurrence and metastasis (2). TSCs have the ability to eliminate chemotherapy drugs from the cell, resulting in its multi-drug resistance (3). TSCs can also activate the DNA mismatch repair system to resist damage induced by radiation (4). In order to reduce tumor recurrence and metastasis, it is necessary to determine the mechanisms of TSC.

There are numerous signaling pathways involved in the formation of TSCs, including the Wnt pathway (5) which involves miRNAs. The mature miRNAs consist of 22 nucleotides, and as negative regulators of gene expression, predominantly recognize the complementary sequences in the 3′ untranslated regions (UTRs) of their target messenger RNAs (6).

95C and 95D cells are NSCLC cell lines, with different metastatic abilities. The effects of miR-544a were studied in 95C and 95D cells in order to reveal the mechanism of GSK3β downregulation, an inhibitory factor of the Wnt pathway (7). The present study aimed to determine the function of miR-544a in the formation of TSCs.

## Materials and methods

### Bioinformatic analysis

**Figure 1:**
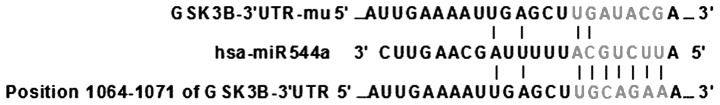
miR-544a can combine with the 3′UTR of GSK3β. miR, microRNA; 3′UTR, 3′ untranslated region.

The miR-544a target gene, GSK3β, was predicted using TargetScan software (http://www.targetscan.org/). The results showed that miR-544a was highly likely to interact with GSK3β (Fig. 1).

### Luciferase assays

Light Switch luciferase assay reagents were obtained from Promega (Promega Corporation, Madison, WI, USA). miRNA negative control (NC) and miR-544a mimic (MC) were transfected together with GSK3β 3′ UTR or GSK3β mutated (MUT) 3′UTR, respectively, into HEK293T cells obtained from the American Type Culture Collection (Manassa, VA, USA) for 24 h according to the manufacturer’s instructions (Promega Corporation). Expression of Firefly (FLUC) and Renilla Luciferase (RLUC) was counted using a luminometer (Promega). Luciferase expression was given as the relative light units (RLUC/LUC) to determine whether GSK3β was the target of miR-544a *in vitro*.

### Transfection

A retroviral vector pBaBe-puro (Addgene, Cambridge, MA, USA) expressing miR-544a was constructed and then inoculated into HEK293T cells for 24 h. The reagents were added to a 1.5 ml Eppendorf tube, including 20 μg PIK, 20 μg expression plasmid, 110 μl ddH_2_O, 250 μl CaCl and 200 μl hepes-buffered saline. The viruses were harvested 24 h after transfection. 95C and 95D cells (American Type Culture Collection) were subsequently infected by these viruses and the cells with highest levels of miR-544a were screened using a puromycin marker. Quantitative polymerase chain reaction (qPCR) was used to identify these cells.

### qPCR

Total RNA was extracted from 95C, 95D, miR-544a-95C and miR-544a-95D cells using TRIzol™ reagent (Invitrogen Life Technologies, Carlsbad, CA, USA) and reverse-transcribed to cDNA using M-MLV reverse transcriptase (Toyobo Co. Ltd., Osaka, Japan). qPCR was performed using a PCR Detection System (Bio-Rad, Hercules, CA, USA) with the use of SYBR^®^ Green I Premix Ex Taq (Takara Bio, Inc., Shiga, Japan). Specific primers for miR-544a were designed by Rui Bo Company (Guangzhou, China). The qPCR reaction was set up as follows: 10 μl 2X SYBR Green I, 0.25 μl 10 pmol/l primers, 1 μl cDNA and ddH_2_O. The reaction protocol included an initial step of 120 sec at 95°C. Each PCR cycle involved denaturation (95°C, 30 sec), annealing (60°C, 35 sec) and extension (72°C, 20 sec) for 40 cycles, and the fluorescence was measured at each cycle. The relative fold change of expression of miR-544a was quantified as 2^−ΔΔ^Ct, where ΔΔCt was Ct (target gene) - Ct (housekeeping gene). Small nuclear RNA U6 was used as a housekeeping gene. The U6 primer sequence was as follows: Forward, 5′-TGGCACCCAGCACAATGAA-3′; and reverse, 5′-CTAAGTCATAGTCCGCCTAGAAGCA-3′.

### Western blotting

95C, 95D, miR-544a-95C and miR-544a-95D cells were lysed by radioimmunoprecipitation assay buffer. The protein concentration was detected by bicinchoninic acid assay (BCA). Protein (20 μg) was loaded onto a 120-g/l SDS-PAGE gel and the proteins were separated at 120 V for 1.5 h. The proteins were then transferred to a polyvinylidene fluoride membrane on ice at 100 V 1 h, and then the membrane was blocked using 5% skimmed milk powder for 2 h at room temperature. The membranes were probed with primary monoclonal rat anti-human GSK3β (Abcam, Cambridge, MA, USA), β-catenin (Abcam), CD133 (Epitomics, Burlingame, CA, USA) and α-Tubulin (Santa Cruz Biotechnology Inc., Santa Cruz, CA, USA) antibodies (1:10,000), as well as a monoclonal rabbit anti-rat IgG secondary horseradish peroxidase (HRP) antibody (Santa Cruz Biotechnology Inc.). Protein expression was quantitatively assessed using an HRP-enhanced chemiluminesence scanner (LAS-4000 mini luminescent imaging analyzer; Fijifilm, Tokyo, Japan)

### Spheroid culture

95C, 95D, miR-544a-95C and miR-544a-95D cells were digested with 0.25% pancreatic enzyme, and 1,000 cells/ml were resuspended in RPMI-1640 serum-free medium. RPMI-1640 media was supplemented with 1X B27 (Gibco-BRL, ), 20 ng/ml EGF (BD Biosciences), 0.4% bovine serum albumin and 4 mg/ml insulin (Sigma-Aldrich). Upon formation of single cell proliferates to spheroids, the spheroids were digested with 0.25% pancreatic enzyme and cultivated as previously described.

### Statistical analysis

One-way analysis of variance with SNK-q test for multiple comparisons was used to analyze the appropriate data using SPSS 15.0 software (SPSS, Inc., Chicago, IL, USA). Data are shown as the mean ± standard deviation. P<0.05 was considered to indicate a statistically significant difference.

## Results

### Validation of miR-544a target gene by luciferase assay

As shown in Table I, luciferase assays revealed that the miR-544a mimic can interact and inhibit the expression of GSK3β 3′UTR (0.52±0.01). The miR-544a mimic could not interact with and inhibit the expression of the GSK3β MUT 3′UTR, and the expression of reporter gene (1.01±0.02) increased (q=491.05, P<0.01).

### Identification of cells stably expressing miR-544a, by qPCR

The expression level of 95C NC and 95D NC was normalized to 1.00±0.00. Following transfection with miR-544a, the miR-544a expression level of 95C and 95D cells was 20.51±0.97 and 15.16±1.38, respectively (F=418.05, P<0.01), among all four groups. As compared with that of the pre-transfection, the expression level was significantly increased (q=19.51 and 14.16, respectively, both P<0.01) (Table II).

### Expression level of proteins of the Wnt pathway by western blotting

**Figure 2:**
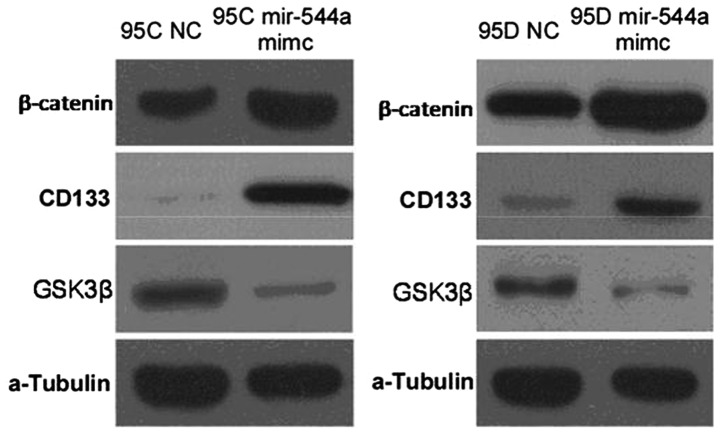
Western blot analysis of protein expression in the Wnt pathway. NC, negative control; mimc, mimic combine; miR, microRNA; 95C, low metastatic human lung cancer cells; 95D, high metastatic human lung cancer cells.

According to the western blot analysis, the level of GSK3β reduced, but that of β-catenin and CD133 increased in 95C and 95D cells transfected with miR-544a. It was therefore concluded that miR-544a activated the Wnt pathway (Fig. 2 and Table III).

### Effect of miR-544a on spheroid formation

**Figure 3:**
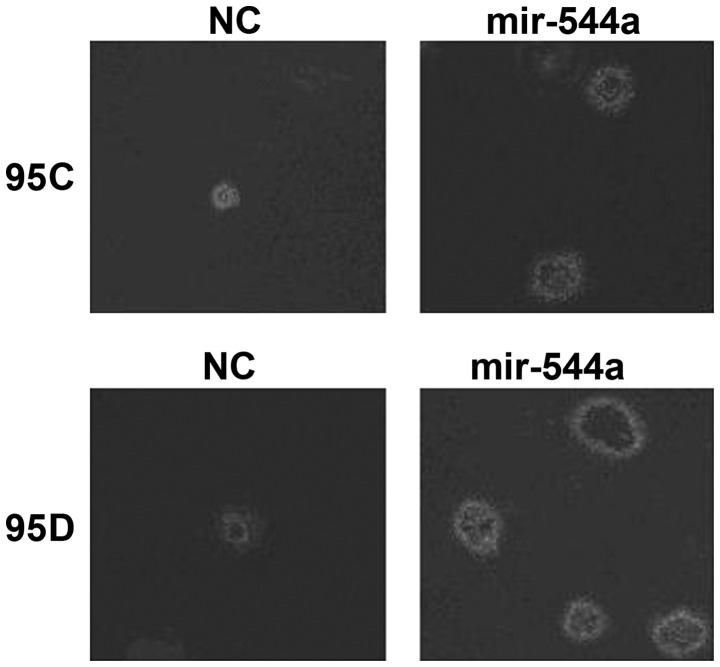
Impact of miR-544a in spheroid formation (magnification, ×200). NC, negative control; mir, microRNA; 95C, low metastatic human lung cancer cells; 95D, high metastatic human lung cancer cells.

Spheroid culture showed that cells stably expressing miR-544a (95C+miR-544a or 95D+miR-544a) had an increased tendency to form tumor spheroids (Fig. 3).

## Discussion

The canonical Wnt pathway is the most well-known and characterized Wnt signaling pathway (8). In the absence of a Wnt ligand binding to its receptor complex, β-catenin is targeted for degradation and the Wnt pathway is shutdown. When the level of β-catenin increases, the Wnt pathway is activated and subsequently the downstream target genes are also activated (9). GSK3β is the most important inhibitory factor in the Wnt pathway. Mutations to or downregulation of GSK3β can lead to the activation of the Wnt pathway and self-renewal (10). While TSCs have an important role in tumor recurrence and metastasis, TSCs have the ability of eliminate chemotherapeutics from cells, therefore resulting in multi-drug resistance of tumor cells (3); TSCs can also activate the DNA mismatch repair system to resist radiation damage (4).

miRNA participates in the development of numerous tumors. miR-544a has been shown to promote tumor invasion and metastasis (11). Other miRNAs, such as miR-34a, miR-107, miR-140 and miR-143 in glioma (12), colon (13), breast (14) and prostate cancer (15), respectively, have been shown to have an important role in TSC formation. Another study has revealed that the level of miR-874 in NSCLC TSCs reduced, leading to the loss of TSC self-renewal and CD133 on the TSC surface (16).

Bioinformatic analyses indicated that miR-544a targeted GSK3β, an inhibitory factor of the Wnt pathway. Luciferase assays validated that miR-544a could interact with and inhibit the expression of GSK3β. Western blot analysis revealed that in cells stably expressing miR-544a, the level of GSK3β was reduced, whereas the expression levels of β-catenin and CD133 were upregulated. To determine the impact of miR-544a in spheroid formation, a spheroid culture was established. It was observed that the cells stably expressing miR-544a had an increased tendency to form tumor CD133-positive spheroids.

In conclusion, miR-544a has an important function not only in tumor invasion and metastasis, but also in TSC formation. Abnormal expression of miR-544a leads to NSCLC self-renewal. Future studies will focus on the mechanism of miR-544a in the formation of TSCs, with a view to novel NSCLC treatment approaches.

## Figures and Tables

**Table I:** Validation of miR-544a target gene by luciferase assay.

Groups	RLUC/FLUC
miR-544a mimic+GSK3β 3′UTR	0.52±0.01
miR-544a mimic+GSK3β MUT3′UTR	1.01±0.02^a^
miR-544a-NC+GSK3β 3′UTR	1.04±0.05^b^
miR-544a-NC+GSK3β MUT3′UTR	1.07±0.03^c^
F-value	201.37
P-value	<0.01

Data represent the mean ± standard deviation, n=9.

a q=491.05;

b q=517.56;

c q=547.93, P<0.01 as compared with miR-544a mimic+GSK3β 3′UTR. 3′UTR, 3′ untranslated region; NC, negative control; miR, microRNA.

**Table II:** Quantitative polymerase chain reaction analysis of miR-544a in 95C and 95D human lung cancer cells.

Groups	2^−ΔΔCT^
95C NC	1.00±0.00
95C+miR-544a	20.51±0.97^a^
95D NC	1.00±0.00
95D+miR-544a	15.16±1.38^b^

Data represent the mean ± standard deviation, n=9.

a q=19.51, P<0.01 as compared with 95C NC;

b q=14.16, P<0.01 as compared with 95D NC.

NC, negative control; miR, microRNA; 95C, low metastatic human lung cancer cells; 95D, high metastatic human lung cancer cells.

**Table III:** Analysis of protein expression in members of the Wnt pathway.

Groups	β-catenin	CD133	GSK3β
95C NC	0.467±0.010	0.000±0.000	0.278±0.013
95C+miR-544a	0.966±0.009^a^	0.660±0.007^a^	0.003±0.003^a^
95D NC	0.656±0.006	0.013±0.006	0.205±0.009
95D +miR-544a	1.489±0.022^b^	0.472±0.007^b^	0.008±0.003^b^
F-value	7.73	3.37	9.43
P-value	<0.01	<0.01	<0.01

Data represent the mean ± standard deviation, n=9.

a q=49.27, 66.00, 2.75, P<0.01 as compared with 95C NC;

b q=83.28, 45.84, 1.95, P<0.01, as compared with 95D NC.

NC, negative control; miR, microRNA; 95C, low metastatic human lung cancer cells; 95D, high metastatic human lung cancer cells.
